# Dentistry in the Northwest

**Published:** 1881-04

**Authors:** J. S. Rice

**Affiliations:** St. Paul, Minn.


### DENTISTRY IN THE NORTHWEST.

BY J. S. RICE, M. D., D. D. S., ST. PAUL, MINN.

ED. REGISTER: In compliance with request I will give the readers of the Register some of my impressions concerning dentistry and some other things in this corner of the Commonwealth.

The dental field in the older and wealthier parts of the country seems to be left in the peaceful possession of the older dentists, while the new generation of dentists rushes forward to these newer fields, with the confidence that their own merits and the growth of population will quickly sweep them into lucrative practice. To such extent have these ideas attained that the immigration of dentists in the Northwest has kept far in advance of the increase in population, and no kind of professional services are at present in more abundant supply than dentistry.

For example; the City of Minneapolis, with a population of forty-eight thousand, has twenty-four dental offices and about thirty dentists all told, and St. Paul, with a few thousand smaller population, is filled to about the same extent, and the quality of the dental talent represented here will compare very favorably with cities farther east or in any part of the country; in fact, it seems that all the dentists who have not been sufficiently rash to go to Europe or California have come to the Northwest. Notable among the established dentists in this city are Drs. Samuel A. Beecher, Geo. O. Lawton and D. C. Price. Dr. Beecher is regarded by the members of the profession as a most skillful and consummate operator, fully up to the modern standard, and conscientious and enthusiastic in his work, and he enjoys a large and very remunerative practice among the best and most appreciative class of patrons. The same can doubtless be said of both Dr. Lawton and Dr. Price. I only take the order of the alphabet in mentioning their names. There are other dentists here of good reputation and ability. There is, however, no dental society and the practitioners, deprived of the privilege of borrowing information from one another, depend on the light of current dental literature or are guided by the principles and landmarks of their past instructions. Fees were in former times very good here, but now the average dentist must be content with the prices that preval in other parts of the country.

The climate of Minnesota has long held the reputation of being quite healthy, but I must confess that I have heard more complaint of lung, throat, nasal and nervous troubles since I have been here than I had ever heard of before during all the rest of my lifetime. Winter commenced early in November, and has held solid up to the present time (March); but, instead of being clear and bright, as these skies are usually said to be in winter, it has been quite cloudy nearly all the time, and there have, of course, been mountains of snow, which drifts to fabulous heights, but there has been no rain to speak of during the last four months. There is a lightness about the atmosphere at times here that is quite oppressive; at such times the smoke and all gases and effluvia hover over the surface of the earth and refuse to ascend and must be breathed, all of which is more or less unpleasant and inimical to health, according as one is constituted or habituated. There are, also, some dental peculiarities here, emanating from the influence of climate, of which I shall be better prepared to speak at another time, after my observations have been more extended. I will, however, venture to state that, according to my observation, there is a similar tendency to low dental vitality, soft teeth and rapid decay, in all extremely cold climates, but more especially those with high altitudes. I reach this conclusion through my observations made in Switzerland and here in Minnesota, and I fancied that I observed the same conditions once during a summer sojourn in the north of Michigan; but the latter, of course, has little altitude. I regret very much my inability to be present at the Mississippi Valley Dental Association.

Fraternally, J. S. RICE.

